# Phase precession through acceleration of local theta rhythm: a biophysical model for the interaction between complex spike cells and theta cells

**DOI:** 10.1186/1471-2202-12-S1-P2

**Published:** 2011-07-18

**Authors:** Luísa Castro, Paulo Aguiar

**Affiliations:** 1 Centro de Matemática da Universidade do Porto, Portugal

## 

Coding information in the hippocampus through phase-precession means that the phase in the theta cycle in which a place cell fires provides information regarding the position of the rat in the place field. There are published models addressing the phase-precession effect but commonly they are grounded in stringent assumptions or ignore the fact that signals in the brain are subject to a high degree of variability and noise. Here we present a biophysical model for phase precession in hippocampal CA1 cells which takes into account the following experimental results: i) there is linear precession through the entire place field of at least 180° of advance for single trials; ii) the firing rate profile through the crossing of the entire place field is Gaussian shaped; iii) phase-precession is present in every trial and not only when the animal has been trained on the circuit; iv) precession occurs during 5 to 6 theta cycles during the crossing of the place field. Our model addresses the interaction between complex spike cells and theta cells in the CA1 field of the hippocampus. The model focuses in the functional block composed of a complex spike cell receiving input from a theta cell which is in turn modulated by the population theta rhythm. The dynamics of the two types of neurons are described by Integrate-and-Fire models with connections being represented as single exponential conductance synapses. Both cells are excited by inputs from the entorhinal cortex (EC) which are modeled using a non-homogeneous Poisson process. The simulated input spike trains representing these inputs are both space modulated and theta rhythm modulated. Phase precession in our biologically plausible model is explained as a consequence of local theta acceleration affecting the complex spike responses. Although all theta cells form a weakly coupled network modulated by the theta rhythm, increased excitation to the pair complex spike cell / theta cell leads to a local acceleration of the average firing frequency of theta cells. The complex spike cell, modulated locally by the theta cell(s) with a rhythm which is slightly faster than the population theta, undergoes phase precession. Only complex spike cells, and associated theta cells, receiving more excitation (inside their respective place field) will exhibit phase precession. Our model implies that rate coding and phase coding in place cells are not produced from independent mechanisms. By its simplicity and reduced assumptions, it can be applied not only to the CA1 precession but also to other hippocampal subfields such as DG and CA3. In fact this model predicts precession in any network with the same architecture and subject to a clocking rhythm, independently of the involvement of the network in spatial tasks.

